# The Impact of Fine-Scale Disturbances on the Predictability of Vegetation Dynamics and Carbon Flux

**DOI:** 10.1371/journal.pone.0152883

**Published:** 2016-04-19

**Authors:** G. C. Hurtt, R. Q. Thomas, J. P. Fisk, R. O. Dubayah, S. L. Sheldon

**Affiliations:** 1 Department of Geographical Sciences, University of Maryland, College Park, MD, United States of America; 2 Department of Forest Resources and Environmental Conservation, Virginia Tech, Blacksburg, VA, United States of America; 3 Applied Geosolutions, Durham, NH, United States of America; Montana State University, UNITED STATES

## Abstract

Predictions from forest ecosystem models are limited in part by large uncertainties in the current state of the land surface, as previous disturbances have important and lasting influences on ecosystem structure and fluxes that can be difficult to detect. Likewise, future disturbances also present a challenge to prediction as their dynamics are episodic and complex and occur across a range of spatial and temporal scales. While large extreme events such as tropical cyclones, fires, or pest outbreaks can produce dramatic consequences, small fine-scale disturbance events are typically much more common and may be as or even more important. This study focuses on the impacts of these smaller disturbance events on the predictability of vegetation dynamics and carbon flux. Using data on vegetation structure collected for the same domain at two different times, i.e. “repeat lidar data”, we test high-resolution model predictions of vegetation dynamics and carbon flux across a range of spatial scales at an important tropical forest site at La Selva Biological Station, Costa Rica. We found that predicted height change from a height-structured ecosystem model compared well to lidar measured height change at the domain scale (~150 ha), but that the model-data mismatch increased exponentially as the spatial scale of evaluation decreased below 20 ha. We demonstrate that such scale-dependent errors can be attributed to errors predicting the pattern of fine-scale forest disturbances. The results of this study illustrate the strong impact fine-scale forest disturbances have on forest dynamics, ultimately limiting the spatial resolution of accurate model predictions.

## Introduction

Forest ecosystem dynamics are characterized by processes of disturbance and recovery across a range of spatial scales, from large catastrophic events including tropical cyclones, fires, and pest outbreaks, to fine-scale forest canopy gap dynamics [1–6]. The spatially and temporally heterogeneous patterns of vegetation structure and fluxes that result from these disturbances present a special challenge to interpretation and prediction [7]. Disturbances episodically alter vegetation structure and create important fluxes of carbon from vegetation to coarse woody debris, litter, and the atmosphere. Recovery following disturbances tends to restore vegetation structure and carbon over longer time scales (decades to centuries) as vegetation regrows and debris decomposes. The complex spatial pattern from a legacy of past events, together with ongoing and potentially changing future events, presents a challenge not only for understanding, but also for prediction.

In light of the need for accurate prediction future forest dynamics and carbon sequestration, how can ecosystem models that account for disturbance processes be evaluated and how does model performance vary across a range of spatial scales? A systematic and quantitative evaluation of model predictions using remote sensing observations is important for assessing the accuracy of model predictions and to facilitate model development [8]. While wall-to-wall estimates of vegetation dynamics and carbon flux at high spatial resolution are rare or non-existent, gridded observations of canopy height change from lidar can potentially be used for model evaluation at spatial scales that range from a lidar footprint (< 1 ha) to large forested domains (> 100 ha).

To ensure the fairest comparison of simulated forest dynamics with observed changes in forest structure, accurate initialization of vegetation in the model is important. Model initialization allows for predicted forest dynamics to reflect the past history of disturbance events. Recent advances linking lidar remote sensing and height-structured forest ecosystem modeling have demonstrated the potential of using these technologies together to reduce uncertainty in initial conditions and thereby constrain model estimates of future carbon stocks and fluxes through the accurate initialization of vegetation structure [9–13]. Previous studies [9,10] used lidar measurements of canopy height to initialize the Ecosystem Demography (ED) model [14–15] at the La Selva Biological Station in Costa Rica, and Hubbard Brook Experimental Forest in Northeastern U.S., respectively. At both study sites, lidar-initialized ED model estimates of aboveground biomass were within <5% of regression-based approaches using field data. Moreover, the resulting predictions of future carbon flux were tightly constrained relative to bracketing alternatives that lacked initialization with data on vegetation structure.

Here, we build on this previous work and use lidar data on vegetation structure collected for the same area at two different times are used to test model predictions of vegetation dynamics across a range of spatial scales at an important tropical forest study site. While multiple measures of vegetation structure are potentially relevant (e.g. biomass, basal area, etc.), we focus on height because both remote sensing and models can directly measure or predict it. Results of this study suggest that fine-scale forest disturbance events limit the spatial resolution of accurate model predictions.

## Methods

### Study Area and Data

The La Selva Biological Station (108259 N, 848009 W) in northeast Costa Rica is a Tropical Wet Forest covering 1600 ha in multiple land-uses including primary and secondary forest [16] It has been a focal site for the development of remote sensing and modeling of vegetation structure in forest ecosystems, especially using the Laser Vegetation Imaging Sensor (LVIS) [9,17–20]. LVIS is a medium altitude waveform digitizing lidar with 10–30 m diameter footprints [21]. The return signal from LVIS represents a vertical record of intercepted canopy surface and is used derive metrics of vertical vegetation structure, including two measures of canopy height: distance above the ground, and distance above Earth’s ellipsoid (i.e. the elevation of canopy top). Estimation of canopy height requires detecting a ground signal via a computer algorithm, and differencing the calculated ground elevation from the canopy top elevation. The latter estimate of canopy height is a potentially preferred metric for calculating canopy height change because it avoids errors associated with locating the ground under forest canopies, but is still sensitive to the threshold used for first-return detection [20]. Research at La Selva has established strong relationships between lidar derived canopy height and field biomass [17–18], and lidar-derived height change and aboveground carbon increment [20]. While land-use history has been shown to be important, above ground biomass does not differ significantly over the relatively small edaphic gradients at this site [17,22].

Two LVIS data collections have been flown at La Selva. The first collection was flown in March 1998 at an altitude of 8 km with a nominal footprint diameter of 25 m. The LVIS footprints were separated by 25 m across the 1 km track and 9 m along track [23]. The second collection was flown in March of 2005 at an altitude of 10 km. The 2 km swath-width had 20 m across track spacing between the 25 m footprints. After both data collections, the vertical waveform was digitized to 30 cm resolution. Canopy Top Height (CTH, RH100) and Canopy Top Elevation (CTE, RH_E_100) were derived for each footprint. For the purpose of model initialization and testing, the footprint data were aggregated to form 1 ha resolution datasets of mean CTH and mean CTE in 1998 and 2005 (for details, see [9,20]).

**Fig 1:**
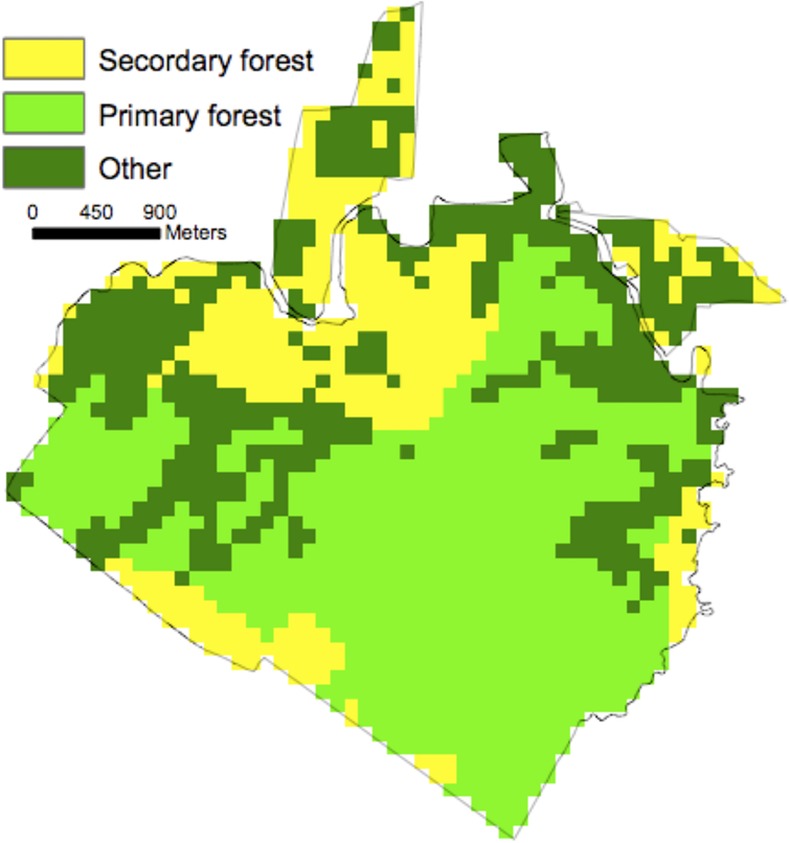
La Selva gridded (1 ha) land-use history classification. Primary denotes natural old-growth vegetation. Secondary denotes vegetation recovering from prior land-use (Organization for Tropical Studies, unpublished data).

**Fig 2:**
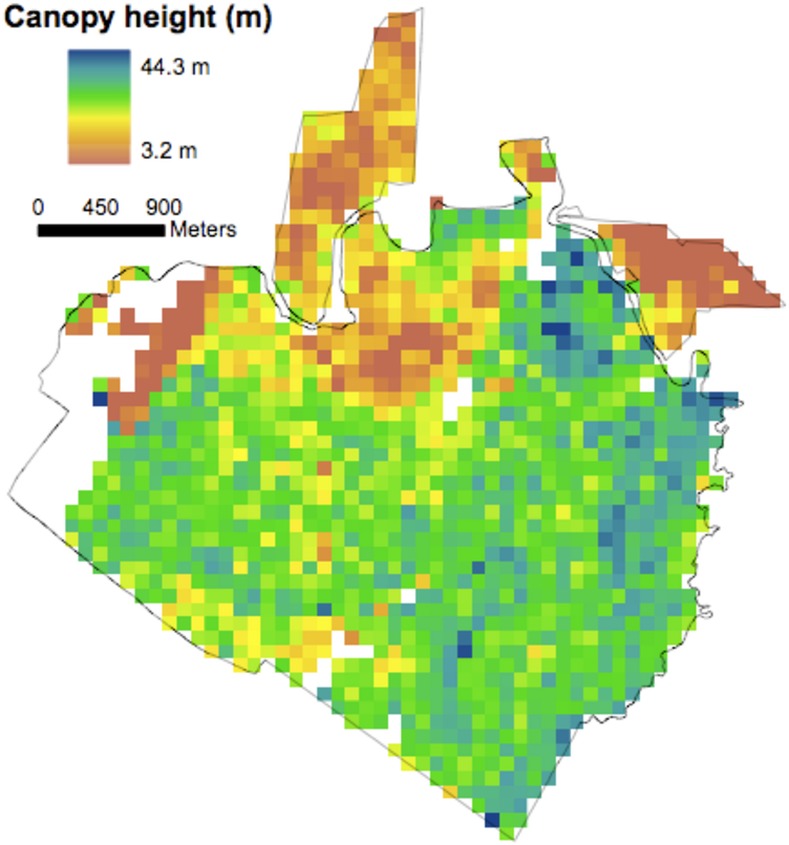
LVIS mean CTH 1998 at 1 ha. [9,20].

**Fig 3:**
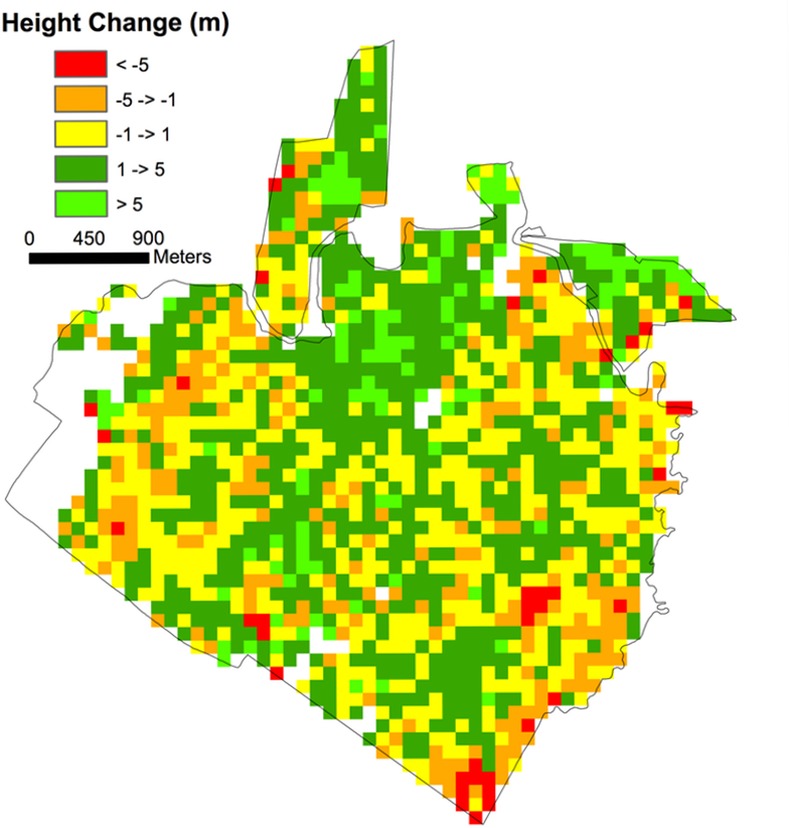
LVIS mean canopy top height change (2005–1998) at 1 ha. Mean canopy height change was determined by differencing elevation of the canopy top in 1998 and 2005 (ΔCTH, see Dubayah et al. 2010).

A total of 1036 ha were classified as forest, 732 old growth (primary) and 304 recovering (secondary) (Fig 1). The mean CTH across the entire domain (primary and secondary) for 1998 and 2005 was 30.6 ± 0.15 m and 31.4 ± 0.14, respectively (1 SE). The mean CTH for primary and secondary forest in 1998 was 32.6 ± 0.11 m and 25.4 ± 0.30 m (1 SE), respectively. Fig 2 shows a map of mean CTH in 1998 at 1 ha resolution. Fig 3 shows a map of LVIS mean canopy top height change (2005–1998) at 1 ha resolution.

### Analysis

Our analysis approach consisted of the following five major steps. 1) Initialize the height-structured ED model [14,15] at La Selva Biological Station using the 1998 lidar canopy top height data. 2) Use the lidar-initalized ED model to predict canopy top height changes across the domain between 1998 and 2005. 3) Compare model predicted changes in mean canopy top height to lidar measured changes in mean canopy top height over the same period. 4) Investigate how the results from the model-data comparison depended on the spatial scale of comparison. 5) Assess corresponding predictions of carbon flux.

To begin, the ED model was initialized with lidar data over La Selva using the initialization protocol presented in [9]. Following that study, 1 ha resolution mean CTH data from 1998 were used in a “look-up table” approach to index corresponding pre-computed ED model estimates of forest structure as predicted to develop through succession at that site. To develop the look-up table, a climatology [24] was used as input to spin-up the model through succession from recently disturbed to old growth conditions, and in the process develop a database relating state variables in the model to mean canopy top height in 0.5 m increments. The 1998 lidar data on mean CTH were used to index the look-up table and initialize the state-variables in the model with corresponding values for each hectare in the domain. Model initialized estimated aboveground biomass across the domain at La Selva was within 1.2% of lidar-derived aboveground biomass (see [9] for more details on the initialization).

**Fig 4:**
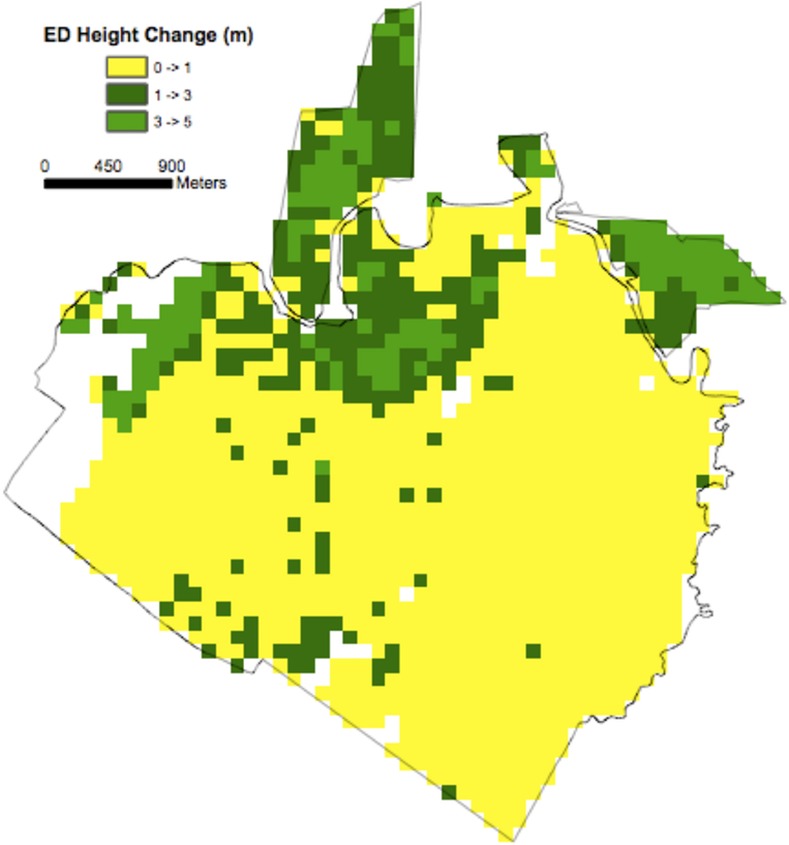
Lidar-initialized ED estimates of mean canopy top height change (2005–1998). The ED model was initialized with 1 ha lidar mean canopy top heights from 1998, and used to predict 1 ha mean canopy top height change in 2005.

Following initialization, ED was used to predict gridded ecosystem dynamics including changes in mean canopy height and aboveground biomass at 1 ha resolution across the domain over the 7-year period between lidar data collections (Fig 4). For consistency, the same climatology used in the initialization was used drive the model forward in time. Hectares observed to be at or above modeled maximum canopy height were predicted to have no change in height, and the corresponding predicted aboveground biomass change was bracketed between zero (dynamic equilibrium) and the net flux estimated for stands when they first reach maximum canopy height.

To test model predictions, predicted gridded changes in mean canopy height were compared to the measured gridded changes using LVIS. To ensure the fairest comparison between the model and lidar data the focus was on 1) hectares with ≥ 20 footprints in both 1998 and 2005 and 2) hectares classified as primary and secondary forest. The dynamics of other land-use types at La Selva, including forest plantations and selective logging, were outside the scope of this study. Model predictions of mean canopy height change were compared to observations based on both lidar measures (ΔCTH and ΔCTE). A standard t-test was used to test for differences between the model predictions and observations. A bootstrap analysis yielded similar results to the t-test.

To investigate the accuracy of model predictions as a function of spatial resolution, both model predicted and observed changes in mean canopy height were aggregated from the 1 ha scale (described above) continuously to the domain scale, and compared modeled and observed changes as a function of spatial scale. Since the domain was not spatially continuous with straight boundaries, neighboring hectares could not be consistently grouped together to form the coarser aggregations. Instead, aggregations were formed by taking 1000 iterations where the hectares are randomly aggregated together at each spatial scale (e.g., for the 2 ha resolution comparison, all hectares were randomly paired and the mean from pairs was used as the canopy height at the 2 ha resolution). The root-mean-square error (RMSE) was calculated for each iteration and a mean RSME was determined for each aggregation scale.

Finally, to help investigate the underlying mechanisms, a second simpler model was used to quantify the expected results in an idealized system in which all aspects of forest dynamics are prescribed. The model [7,11] is a highly simplified spatial stochastic forest simulator that represents an idealized forest landscape on a gridded horizontal plane where each grid cell was the approximate size of a canopy tree (e.g. 10 m; as implemented in the ED model). At each time step, height growth occurred as a saturating function of height in the grid cell, and disturbance followed a power-law size-frequency relationship typical of forest ecosystems [7, 25–26]. The model was first used to create a hypothetical forest with a range of successional states, analogous to the heterogeneity at La Selva. Then it was run an additional 7 years, comparable to the time between successive lidar measurements. This served as a reference case. An additional set of 7-year simulations were run that used the same initial state as the reference case, but in which the complex spatial pattern of disturbance was replaced with a uniform rate, and potential errors in growth could be investigated and compared to results obtained using ED.

## Results

At the domain scale, lidar-initialized model predictions of canopy height change compared favorably to observed changes in canopy (Table 1). Averaged over the domain, the model prediction of mean canopy height change was 0.53 ± 0.04 m. This result was close to (within < 0.5 m), in between, and significantly different than the observed changes in mean canopy height based on the two lidar methods (ΔCTH 0.85 ± 0.09 and ΔCTE 0.37 ± 0.07). Analogous results were obtained for the subset of primary forest, where model predictions of canopy height change (0.04 ± 0.01 m) and observations of canopy height change (ΔCTH 0.44 ± 0.09 m and ΔCTE -0.32 ± 0.06 m) were as expected lower than for the domain as a whole. Over secondary forest, predicted and observed height changes were larger, also as expected. Here, model predictions of canopy height change (1.71 ± 0.09 m) were closer to observations and significance depended on lidar method (ΔCTH 1.84 ± 0.18 m, ΔCTE 2.08 ± 0.13 m). Stratifying by canopy height and considering only patches below max canopy height simulated by the ED model (1 ha mean 2005 CTH < 27.5 m), height changes were larger still, and model predictions of canopy height change (2.71 ± 0.04 m) compared even more favorably to observations and were not significantly different than data based on either lidar method (ΔCTH 2.63 ± 0.22 m and ΔCTE 2.69 ± 0.15 m). An analagous statistical result was found for the subset of these patches classified as Secondary; the subset classified as Primary could not be tested due to small sample size.

**Table 1:** Mean canopy height change (2005–1998).

Forest Type	N	Mean ΔCTH	Mean ΔCTE	Mean ΔED Height
All	886	0.85 ± 0.09	0.37 ± 0.07	0.53 ± 0.04*†
Primary	629	0.44 ± 0.09	-0.32 ± 0.06	0.04 ± 0.01*†
Secondary	257	1.84 ± 0.18	2.08 ± 0.13	1.71 ± 0.09†
All–height < 27.5 m	172	2.63 ± 0.22	2.69 ± 0.15	2.71 ± 0.04
Primary–height < 27.5 m	13	3.58 ± 1.03	0.12 ± 0.4	2.13 ± 0.08^NA^
Secondary–height < 27.5 m	159	2.55 ± 0.23	2.90 ± 0.15	2.76 ± 0.04

N represents the number of hectares with ≥ 20 lidar footprints in both 1998 and 2005.

*ΔCTH* is the change in mean canopy height determined by differencing lidar vegetation height in 1998 and 2005.

*ΔCTE* is the change in mean canopy height determined from changes in lidar canopy top height elevation.

*ΔED Height* is the lidar-initialized ED estimate of changes in mean canopy height.

All uncertainty values are ± 1 S.E.

A statistically significant difference between the ED estimate and ΔCTH is marked by * and ΔCTE is marked by †.

NA indicates statistical test not preformed due to small sample size.

Statistical significance was assessed at the 0.05 level using both t-test and bootstrap methods; both methods agreed with one another.

*NA* indicates statistical test not preformed due to small sample size.

**Fig 5:**
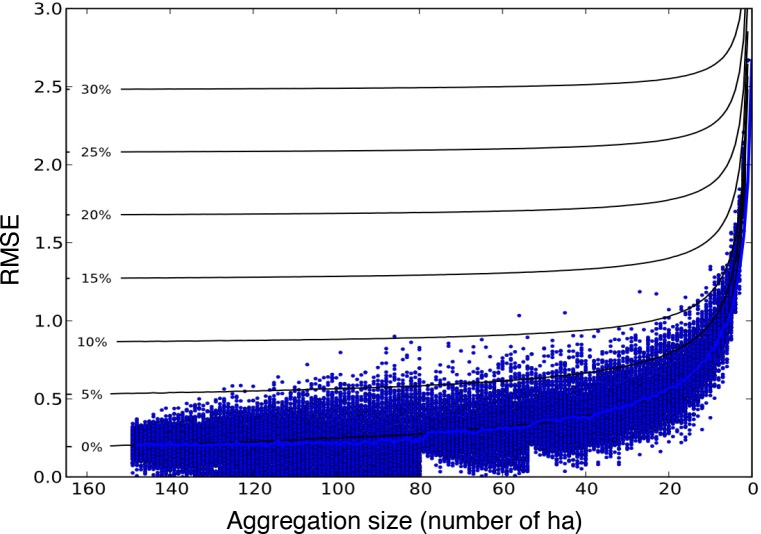
Root mean squared error between the ΔCTH lidar measurement and the ED model prediction as a function of spatial scale. The model-data comparison at coarse scales (i.e. >50 ha) has relatively low error (RMSE). The error increases rapidly as the spatial resolution of comparison increases. Contours denote the additional expected average RMSE using the simulator at each scale assuming potential systematic bias errors in predicted growth or mortality. Only results with no systematic bias in growth or mortality produce results of similar magnitude as those predicted by ED.

The spatial scale of comparison had a strong influence on the accuracy of model predictions (Fig 5). At the coarsest scale analyzed, 150 ha, the predicted canopy height change was in close agreement to observed (RMSE<0.25 m). Prediction error increased non-linearly with increasing spatial resolution. At 20 ha, prediction error was approximately twice the result at the coarsest scale, and at 1 ha the prediction error was nearly ten times higher (RMSE<2.69 m).

**Fig 6:**
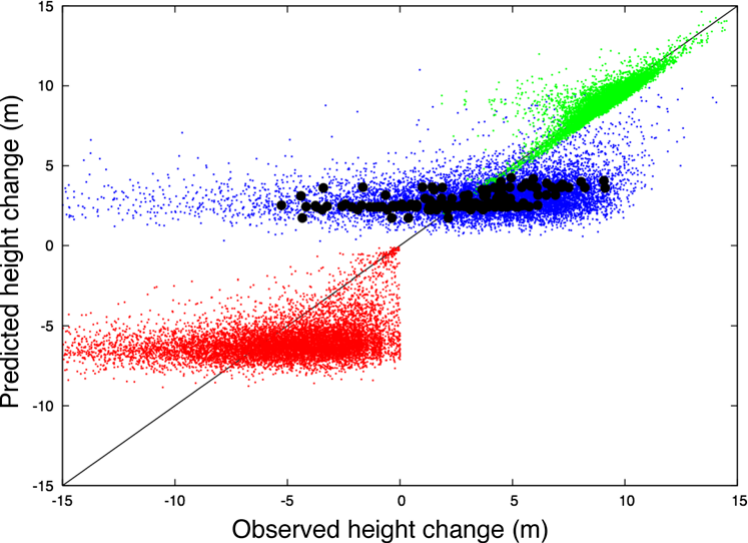
Simulator estimates of mean canopy top height change at 1 ha resolution (2005–1998, y-axis) compared to the corresponding observed mean canopy top height change (x-axis). Solid-line 1:1 line. Green symbols: simulator predicted height change due to growth. Red symbols: simulator predicted height change due to mortality. Blue symbols: simulator predicted net height change. Black symbols: lidar- initialized ED prediction vs. ΔCTH. Replacing the complex pattern of spatial disturbances with a uniform pattern in the simulator results in fine-resolution errors in predicted mortality that propagate to errors in net predicted height change similar to that predicted by ED.

Analyses using the second simpler model (simulator) indicate these errors are likely due to the fine-scale spatial pattern of disturbance per se. Model error contours generated using the simulator (Fig 5) illustrate that a simulation with no error in growth or mortality rates (0% contour), but which failed to capture the complex spatial pattern of disturbance, produced results similar to those found using ED. Systematic bias in either of the two other potential factors of growth or mortality rates (>0% contours) did not produce results such as this, but instead increase the expected RMSE at all spatial scales. Deconstructing the net height change at 1 ha resolution into predicted growth and mortality components (Fig 6) illustrates that application of uniform disturbance rates at this scale results in error in the mortality component which propagated to error in the predicted net height change similar to that predicted by ED. These errors in predicted net height change occurred despite accurate predictions of growth.

Finally, these results have major implications for carbon as well as height due to the strong association between vegetation height and biomass. At the domain scale, lidar-initialized ED predicted aboveground carbon flux at La Selva was 0.019–0.053 kg C m^-2^ yr^-1^ (upper and lower bounds). Within the secondary forest, the predicted carbon flux was larger, and ranged between 0.056–0.072 kg C m^-2^ yr^-1^ for all hectares and as expected was higher still at 0.103 kg C m^-2^ yr^-1^ the subset less than 27.5 m (Table 2). The ED predicted carbon flux in the secondary forest was similar whether the change was predicted from a 1998 lidar initialization, or determined by initializing the carbon stocks with lidar in 1998 and 2005 separately and differencing the stocks (Table 2). These ED-based carbon flux estimates were similar to and inclusive of estimates of aboveground flux made by differencing empirical biomass maps (“LVIS differenced” method, Table 2). The scale dependency of prediction error for carbon was also strong and analogous to that for height.

**Table 2:** Average net above ground carbon flux on secondary land (kg C m^-2^ yr^-1^).

	All heights & all # of footprints			All heights & ≥ 20 footprints			Height < 27.5 m & ≥ 20 footprints	
Estimation method	N	Lower bound	Upper bound	N	Lower bound	Upper bound	N	Estimate
ED predicted	334	0.056 (0.002)	0.072 (0.002)	257	0.060 (0.003)	0.073 (0.02)	111	0.103 (0.002)
ED differenced (*ΔCTH*)	334	0.042 (0.004)	0.11 (0.014)	257	0.045 (0.004)	0.11 (0.016)	111	0.089 (0.012)
ED differenced (*ΔCTE*)	334	0.048 (0.004)	0.11 (0.013)	257	0.060 (0.004)	0.126 (0.013)	111	0.108 (0.007)
LVIS differenced (*ΔCTH*)	334	0.070 (0.006)	0.070 (0.006)	257	0.069 (0.006)	0.069 (0.006)	111	0.091 (0.011)
LVIS differenced (*ΔCTE*)	334	0.071 (0.04)	0.071 (0.04)	257	0.078(0.005)	0.078 (0.005)	111	0.110 (0.007)

Lower and upper bounds represent the assumptions about carbon increment in hectares with height > = 27.5 m. The bounds do not apply to lidar measures but are presented alongside the model bounds. Means with standard error (SE). The SE is from the aggregation of the 1 ha data and does not represent error in the 1 ha level estimates.

## Discussion

Forest disturbances occur across a wide range of spatial and temporal scales, and are known to influence both the structure and dynamics of forest ecosystems. But much remains unknown about the pattern and predictability of disturbance events and their consequences. While large dramatic events such as hurricanes, fires etc, have garnered the attention of remote sensing and modeling communities, and forest gap dynamics have long been known to impact forest dynamics [27,28], relatively less attention has been given recently to characterizing and understanding the pattern and impact on model prediction of these relatively fine-scale events which are typically much more numerous. Here, advances in high-resolution modeling and remote sensing are combined to test model predictions of forest dynamics over an intensively studied field site. Results confirm the importance of fine-scale disturbance events to forest dynamics, and more profoundly demonstrate a scale-dependence of predictability that is driven by the pattern of fine-scale stochastic disturbance events.

The capacity to accurately predict height change at high spatial resolution in this study was ultimately limited by the ability to predict the pattern of fine-scale disturbances. In principle, model prediction errors could have been caused by errors in predicted growth, mortality, or both. However, systematic bias in either growth or mortality would not produce the spatial-scale dependent errors found here, because bias would produce error at all scales (Fig 5). Previous studies have shown that disturbances typically follow a power-spectrum distribution [7, 25–26], and are highly variable at 1 ha resolution. Failure to represent this heterogeneity produces the scale-dependent pattern of errors observed with ED. From a practical point of view, accurately modeling vegetation dynamics at 1 ha resolution will require models able to deterministically predict the heterogeneity of disturbance events in each hectare, which is beyond current model capabilities. However, highly accurate model predictions of vegetation dynamics at coarser scales, in this case >20–50 ha, are possible as disturbance rates were less variable and better characterized by an average rate at these scales.

We hypothesize that the finding of scale dependence in the predictability of vegetation dynamics found at this site is common to other forest systems. Complex fine-scale disturbance patterns are common in forests, but are not represented in current prognostic approaches. Furthermore, we expect that the steepness of the scale dependence will vary between different disturbance regimes. In a forests dominated by relatively frequent, small, and unclustered disturbance events (i.e. steep power-law size-frequency relationship of events), we expect relatively high resolutions of predictability will be possible because the vast majority of disturbance events repeat within a small units of area. In contrast, in forests dominated by more infrequent, larger, and clustered events (i.e. flatter power-law size-frequency relationship of events), we expect coarser limits to accurate predictions because the unit area required for a vast majority of events to repeat would be larger.

This study focused on quantifying vegetation dynamics in units of height, native to both lidar remote sensing and the height-structured ecosystem model used, and thereby providing a direct linkage between data and model. However, analogous results were also obtained for carbon due to the strong relationship between height and carbon. Quantitatively, two different empirically based estimates of the magnitude of carbon flux at this site are available and appear inconsistent; the results from this study agree with one, and are lower than the second. The magnitude of carbon flux predicted by the model are consistent with the approach using the site-specific empirical relationship between lidar height and aboveground carbon stocks [9,17] to calculate the carbon flux by differencing biomass maps from the initial and ending period (Table 2). However this difference-based estimate is 37–77% lower than the carbon flux on secondary forests at the same site using an alternative approach using repeat lidar to estimate flux directly [20]. Reconciling the differences between these alternative approaches to carbon flux is beyond the scope of the present study, and it is important to note that they do not diminish the results found here in native height units or the finding of scale dependent predictability.

With ever increasing advances in technology (e.g. remote sensing, computing, etc.), we live in an increasingly data-rich world. But the capacity to measure and simulate vegetation dynamics at increasingly higher resolutions does not automatically confer increased predictability at ever higher resolutions. As spatial resolution increases, at some level predictability will require corresponding advances in process representation. In particular, achieving high predictability at the highest spatial resolutions investigated here will require both data at these resolutions and new process representation in models to predict the complex spatial pattern of forest disturbance at these scales. Meanwhile, the finding of the spatial-scale dependent accuracy of model results with the current modeling approach implies there are corresponding limits to the spatial resolution of the use of these model predictions in applications and potential future land-management decisions.
